# A Novel Homozygous Variant in the CHRNE Gene in 2 Siblings with Congenital Myasthenic Syndrome

**DOI:** 10.1177/2329048X231216432

**Published:** 2023-11-28

**Authors:** Cassie Chan, Lucy Emery, Caroline Maltese, Ashutosh Kumar, Ermal Aliu, Sunil Naik, Dustin Paul

**Affiliations:** 1 Department of Neurology, Penn State Health Milton S. Hershey Medical Center, Hershey, PA, USA; 2 12310 Penn State Health College of Medicine, Hershey, PA, USA; 3 Department of Pediatrics, Penn State Health Milton S. Hershey Medical Center, Hershey, PA, USA; 4 Department of Genetics, 12311 Penn State Health Milton S. Hershey Medical Center, Hershey, PA, USA

**Keywords:** congenital myasthenic syndrome, CMS, CHRNE, pediatric, neuromuscular

## Abstract

Cholinergic receptor nicotinic epsilon (CHRNE) subunit mutations cause postsynaptic type of congenital myasthenic syndrome either as a primary acetylcholine-receptor deficiency or abnormal channel kinetics in the receptor. We report a novel homozygous variant (c.322C > T, p.Pro108Ser) in the epsilon subunit causing primary acetylcholine-receptor deficiency in two siblings. Two siblings presented with fatigable weakness. Both siblings had whole exome sequencing showing a homozygous variant (c.322C > T, p.Pro108Ser) of unknown significance in the epsilon subunit. Electromyography/nerve conduction study with repetitive nerve stimulation on one sibling showed a defect in neuromuscular junction transmission. Pseudoephedrine and fluoxetine for suspected slow-channel congenital myasthenic syndrome yielded no improvement. A trial of pyridostigmine led to clinical improvement. Given the clinical presentation, consanguinity, homozygous genetic variant, and response to pyridostigmine, we rationalize the homozygous variant (c.322C > T, p.Pro108Ser) in cholinergic receptor nicotinic epsilon subunit causes the primary acetylcholine-receptor deficiency congenital myasthenic syndrome.

## Introduction

Congenital myasthenic syndromes (CMS) are inherited disorders that cause dysfunction of neuromuscular transmission at the motor endplate.^1–3^ Presentation of CMS can vary in terms of age of onset, initial symptoms, and treatment, but these syndromes typically share the clinical feature of fatigable muscle weakness.^2^ The spectrum of CMS presentations is largely dependent on the underlying genetic defect in the neuromuscular junction (NMJ).^1^ At the NMJ, acetylcholine is released from the presynaptic neuron into the synaptic cleft where it binds to the acetylcholine receptor (AChR).^1^ Then, ion channels open, and sodium flows into the cells.^1^ When the ion channels close, acetylcholine dissociates and is degraded by acetylcholinesterase or cleared by diffusion.^1^ If the endplate depolarizes, then muscle contraction occurs. The amplitude of the endplate action potential is a function of the number of synaptic vesicles released by a nerve impulse and the quanta of neurotransmitters released. Therefore, presynaptic, synaptic, or postsynaptic dysfunction could lead to impaired neuromuscular transmission, causing CMS.^2,3^

Genetic defects most commonly affect the postsynaptic acetylcholine receptor, but mutations in presynaptic proteins, defects in endplate development, mutations in the synaptic basal lamina, and defects in protein glycosylation are also involved.^1,4^ Characterization of CMS is based on inheritance and localization of defects at the NMJ.^1,4^ CMS has a wide range of severity, from mild, phasic weakness, to permanent muscle weakness, respiratory insufficiency, and even death.^5^ Presenting symptoms, age of onset, and treatment response of CMS vary based on underlying genetic defect. Diagnosis of CMS involves repetitive nerve stimulation (RNS) findings of electrodecremental motor response showing a greater than 10% decrease from the first to the fourth stimulation.^1,6^ Diagnostic genetic testing includes panels that cover CMS genes or broader testing such as whole exome/genome sequencing. To date, there are more than 30 genes known to cause CMS.^1,4,6,7^ Mutations in the cholinergic receptor nicotinic epsilon (CHRNE) subunit gene cause acetylcholine-receptor deficiency and abnormal channel kinetics.^5^ They are the most frequent genetically diagnosed cause of CMS, accounting for 30%-50% of cases.^5,8^ In this study, we present clinical and genetic findings in 2 siblings who have a homozygous variant of unknown significance, c.322C > T; p.Pro108Ser, on exon 4 of the CHRNE gene (NM_000080.4).

## Case Report

Two full siblings, a 17-year-old female and a 9-year-old male, presented for evaluation of proximal muscle weakness and fatigable ptosis. Both siblings were products of a consanguineous marriage and have no family history of similar presentations.

The girl attained all milestones on time. Her parents first noticed that she had bilateral ptosis around 4-5 years of age with associated weakness in her extremities after exertion. The girl described worse symptoms upon waking that improved with walking, but the ptosis worsened towards the evening.

The boy sat at around 8 months old, but he did not stand until 18 months. At four years of age he developed muscle fatigue and a stooped posture. Around that time he also developed bilateral fatigable ptosis.

On initial examination, both siblings had bilateral fatigable ptosis which worsened with sustained upward gaze and fluctuating muscle weakness with fatigability on motor examination. Additionally, the boy had accentuated lumbar lordosis and bilateral knee flexion and plantar flexion contractures.

Both underwent genetic testing that revealed a homozygous missense variant of uncertain clinical significance, c.322C > T; p.Pro108Ser, in the CHRNE gene. This missense mutation has not been observed in public databases (gnomAD, LVOD, Clinvar). It is predicted to be deleterious by several in silico algorithms (CADD, mutationtaster, MutPred, PROVEAN, Polyphen-2), and Proline at 108 amino acid position is highly conserved through species. Since the variant was in the CHRNE gene, which codes for the epsilon subunit of the acetylcholine receptor, there was concern for a slow-channel CMS. As a result, the boy was initially treated with fluoxetine, without response. The girl was previously treated with pseudoephedrine without response.

Both siblings began a trial of pyridostigmine and showed dramatic improvement, contrary to slow-channel mutation cases that typically worsen with acetylcholinesterase inhibitors. The girl reported overall improvement in her bilateral ptosis with increased activity tolerance. Repeat neurological examination showed improvement in bilateral ptosis and muscle weakness with fatigability. The boy also reported increased activity throughout the day and his gait posture and exercise tolerance improved.

EMG/NCS with slow RNS on the girl was performed 48 h after stopping pyridostigmine. Repetitive CMAPs were not appreciated. RNS at 3 Hz of the left facial nerve demonstrated a decrement of 61.9% immediately post-exercise. RNS of the left spinal accessory nerve demonstrated a baseline decrement of 10.8%. Given the decrement, the results suggested a post-synaptic NMJ transmission defect, which correlates with the improvement of symptoms with pyridostigmine.

## Discussion

The adult nicotinic AChR is made up of four transmembrane subunits: α, β, δ, and ε subunits.^2^ About three-quarters of CMS cases are secondary to mutations in the AChR subunits.^9,10^ Mutations in AChR subunit genes can either diminish expression of AChR or change its kinetic properties or decrease or increase the response to acetylcholine.^11^ The CHRNE gene mutations compose up to half of the known gene mutations in CMS patients.^12^

Postsynaptic CMS resulting in primary AChR deficiency is caused by biallelic loss of function variants which do not cause kinetic alterations, in any of the AChR subunits.^2,13^ Primary AchR deficiency is characterized by 10%-30% reduction in the number of functioning AChR and dysfunctional receptor folding.^2^ These patients present with varying phenotypes, but most patients present with eyelid ptosis, limited ocular movements, fatigable weakness, and moderate-to-severe limb muscle weakness.^1,2^ For many patients with mutations in the epsilon subunit, there is benefit from cholinergic agonists, and some patients receive additional benefit from salbutamol.^11^ For patients with mutations in non-epsilon subunits, symptoms are more severe, and mortality is high in infancy or early childhood.^1^

### Slow-Channel CMS

Slow-channel CMS typically results from heterozygous gain of function variants, in the ligand-binding (thus increasing affinity for acetylcholine) or pore domains (enhances receptor activation after agonist binding) of the AChR, resulting in prolonged synaptic currents and action potentials.^1,13,14^ Rarely it can have a recessive inheritance pattern.^15^ Mutations in CHRNA1 and CHRNE are most common for AChR kinetic abnormalities.^2^ The prolonged opening of the AChR causes desensitization of the AChR and depolarization block^2^ leading to calcium accumulation in the postsynaptic region,^16^ leading to endplate myopathy. Patients with slow-channel CMS usually present in early life with more severe cases presenting in the neonatal period.^1,2^ Clinical presentation includes severe involvement of the cervical, scapular, and dorsal forearm muscles.^1^ Ptosis and ophthalmoparesis can occur to a lesser degree than in primary AChR deficiency.^1,2^

### Fast-Channel CMS

Fast-channel CMS is caused by a kinetic altering fast-channel variant in one allele of an AChR subunit, accompanied by a null or low-expressor variant, or rarely by another fast-channel variant, on the other allele.^16^ With fast-channel variants, channel openings are brief with secondary decreased postsynaptic depolarization and failure to trigger muscle action potentials.^2^ Unlike primary AChR deficiency and slow-channel CMS, there are no clinical clues to discern a fast-channel CMS. Clinical consequences are based on pathogenic mutation location, with more severe pathology occurring when the mutation is located at or near the agonist binding site,^17^ affects coupling of agonist binding to gaiting,^18^ or is located in a transmembrane domain.^19^ The phenotype is less severe when the mutation is in the long cytoplasmic loop of the epsilon subunit.^20,21^

Diagnosis of CMS is made based on many or all of the following: early age of onset, fatigable weakness (especially of the ocular and other cranial muscles), a decremental electromyographic response - defined as greater than 10% decrease of the amplitude or area of the fourth, compared to the first, evoked compound motor action potential (CMAP) on RNS, or an abnormal single-fiber EMG (SFEMG) response, and positive response to pharmacological treatment.^1,2^ Genetic diagnosis of CMS is more complex with more than 30 causative genes identified.^2^ Phenotypic features such as age of onset, distribution of weakness, electrodiagnostic findings, and response to treatment, can help tailor genetic screening. Next-generation sequencing has facilitated the discovery of new CMS genes and their corresponding phenotype.^22^

Current therapies for CMS include acetylcholinesterase inhibitors and cholinergic agonists (eg, pyridostigmine and amifampridine, respectively), long-lived open-channel blockers of the acetylcholine receptor ion channel (eg, fluoxetine and quinidine), and adrenergic agonists (eg, salbutamol and ephedrine). Pyridostigmine acts by inhibiting acetylcholinesterase in the synaptic basal lamina, which increases the number of acetylcholine receptors activated by a single quantum. Amifampridine increases the number of acetylcholine quanta released by each nerve impulse. Alone, and especially in combination, these agonists increase the amplitude of the endplate potential and thereby meet the safety margin of neuromuscular transmission. Therefore, they are beneficial in patients with endplate acetylcholine-receptor deficiency and those with fast-channel syndromes (although effect may decrease with time in these patients), in which the safety margin of transmission is compromised by a decreased synaptic response to acetylcholine and an abnormally fast decay of the synaptic current. On the other hand, drugs that increase acetylcholine levels can have the opposite effect in slow-channel CMS.^2^ Fluoxetine and quinidine are long-lived open-channel blockers of the acetylcholine receptor used in the treatment of the slow-channel syndrome. They prevent a depolarization block and desensitization of acetylcholine receptors at physiological rates of stimulation and mitigate the cationic overloading.

A case report described benefit with albuterol in 2 patients with CMS due to epsilon-subunit mutations.^11^ In one patient treated with albuterol sulfate, limb strength improved greatly within a few weeks but had no change in the ophthalmoplegia or facial weakness. A second patient was treated with a similar dosage of albuterol sulfate and received marked improvement in limb strength within several weeks.

Given the electrodecremental response on RNS and our patients positive response to pyridostigmine, their mutation in CHRNE gene can be classified as a form of fast-channel CMS or CMS type 4B.

## Conclusion

Given homozygosity, allelic frequency, conservation, and in silico predictions, this is likely an autosomal recessive presentation, consistent with AChR-e type of CMS. This expands on current knowledge of the effects of pathogenic CHNRE variants and hypothesizes that this specific variant is pathogenic for CMS. CHRNE gene mutations have been associated with CMS. Patients typically present with proximal weakness and fatigable ptosis as seen in both of these siblings. Given that the patients improved with pyridostigmine and had no response to fluoxetine and pseudoephedrine, c.322C > T(p.Pro108Ser) variant in homozygous state causes a primary AChR deficiency type of CMS.
